# Cross-sectional study on hearing loss and auditory reaction time before and after spinal anesthesia with marcaine 0.5% in patients undergoing elective surgery

**DOI:** 10.1016/j.amsu.2020.10.046

**Published:** 2020-11-01

**Authors:** Sepideh Vahabi, Parvin Veiskarami, Mehdi Roozbahani, Shahrzad Lashani, Behrouz Farzan

**Affiliations:** a Department of Anesthesiology, Faculty of Medicine, Lorestan University of Medical Sciences, Khorramabad, Iran; b Department of Audiology, Faculty of Medicine, Lorestan University of Medical Sciences, Khorramabad, Iran; c Department of Motor Behavior, Borujerd Branch, Islamic Azad University, Borujerd, Iran; d Student Research Committee, Faculty of Medicine, Lorestan University of Medical Sciences, Khorramabad, Iran

**Keywords:** Spinal anesthesia, Hearing loss, Auditory reaction time, Marcaine, Surgery, Blood pressure

## Abstract

**Background:**

Hearing loss is a rarely reported complication of spinal anesthesia. The purpose of this study is to assess the effects of 0.5% Maracine (bupivacaine) on hearing threshold and auditory reaction time before and after spinal anesthesia among patients undergoing elective surgery.

**Materials and methods:**

This is a descriptive cross-sectional study performed on 60 patients undergoing elective surgery with ASA Class II and II anesthesia (0.5% bupivacaine) at Khorramabad Nursing Home**.** After obtaining consent from the patients, audiometry and tympanometry tests were performed using AZ80 and Madsen otoflex tympanometer and related findings including the presence or absence of hearing loss at various frequencies, before and after the surgery, were noted in a form for each patients along with their demographic data. SPSS 21 was used for statistical analysis and the data were analyzed using descriptive statistics and chi-square inferential tests.

**Results:**

At low frequencies of 250 and 500 Hz, no significant difference in pre- and postoperative hearing threshold in the right ear (P > 0.05) was seen, but at frequencies above 500 Hz, the hearing threshold was significantly decreased after surgery, (P < 0.05). In the left ear at 250, 1000, 3000, and 8000 Hz, there was no significant difference (P > 0.05) between pre- and postoperative hearing threshold. The results of this study showed that the preoperative hearing threshold for men and women did not differ and the auditory threshold and auditory response time after surgery did not differ between the two sexes (P > 0.05). Similarly, the difference was not correlated with the age and the levels of anesthesia (P > 0.05). The results also showed that changes in mean arterial blood pressure (MAP) and heart rate above 30% of baseline were also not correlated with hearing loss (P > 0.05).

**Conclusions:**

The results showed that at certain frequencies, hearing loss was observed in both ears after spinal anesthesia with 5% Marcaine, but this hearing loss was not related to age, sex, and spinal anesthesia level. The results also showed that changes in mean arterial blood pressure (MAP) and heart rate above 30% of baseline did not correlate with hearing loss.

## Highlights

• Hearing loss is a rarely reported complication of spinal anesthesia.
• Hearing loss in both ears is observed after spinal anesthesia with 5% Marcaine.
• Changes in MAP and heart rate greater than 30% of baseline is not associated with the hearing loss.

## 1 Introduction

Spinal anesthesia is a subdivision of directional or regional anesthesia, together with epidural anesthesia, known as the central regional block [1]. It is used in surgical anesthesia (as a supplement to general anesthesia) and midwifery and postoperative pain control [2]. Spinal anesthesia is the most common method of elective cesarean section due to reduced need of intubation and anti-depressants [3]. Provided the nature of the surgery, spinal anesthesia favors patients with respiratory illness, such as chronic obstructive pulmonary disease, reducing the need of intubation and mechanical ventilation [4]. Additionally, it is also useful in patients who have difficulty with endotracheal intubation due to anatomical abnormalities [3,5]. However, complications such as hypertension, bradycardia, postoperative headache, nausea, diplopia, tinnitus, infection transmission, discopathy, nerve damage, urinary retention and genital injury are reported [6,7]. One of the scarcely reported complications associated with spinal anesthesia include unilateral or bilateral sensorineural hearing loss [8]. The complication is not known to have clear etiology however, leakage of cerebrospinal fluid (CSF) as a result of dural puncture can be a possible cause of hearing impairment, similar to postdural puncture headache [9]. A drop in CSF pressure that is received in inner ear, causing endolymphatic hydrops, leads to the displacement of hair cells of basal membrane and vestibular-cochlear nerve dysfunction [10]. The incidence of these complications varies from 3 to 92%. Low frequency hearing loss can occur between 125 and 1000 Hz and usually occur within 2 days after spinal anesthesia [11]. However, some studies reported hearing loss lasting longer than 7 months [12] or more than 2 years after spinal anesthesia [13]. This risk is high in elderly patients as cochlear function decreases with age.

Bupivacaine is an amide that has 10-min slow onset and is more effective with isobaric forms [14]. It is suitable for surgeries that last for 2–2.5 h [15,16]. This is comparable to tetracaine, however, tetracaine shows a deeper motor block and increases the duration of tensile strength [17,18]. Available forms include concentrations of 0.5% and 0.75% and dextrose 8.25%. Bupivacaine and lidocaine can be used interchangeably [19].

The variability of the solutions used for spinal anesthesia appears to cause significant differences in the distribution of anesthetic drugs within the spinal cord, which can have important effects on the extent of onset and duration of the anesthetic block, as well as the side effects [20,21]. Studies have shown that hypertensive bupivacaine initiates sensory block for cesarean section and is less likely to convert anesthesia block to general anesthesia [22,23].

The aim of this study is to investigate the effects of spinal anesthesia, Marcaine 0.5% (bupivacaine) on hearing impairment among patients undergoing elective surgery.

## 2. methods

2

In this descriptive cross-sectional study, all the patients referred for elective surgery meeting the requirements of American Anesthesiologists Association (ASA) Class I and II anesthesia I at Khorramabad Nursing Home were enrolled. The inclusion criteria of the study included: patients aged 19–45 years undergoing elective surgery with ASA Class I and II, patients with normal otoscopic examination before surgery, hearing threshold above 30 dB and type A tympanogram, without the history of otitis media, meningitis, systemic disease, mumps, ear surgery or the intake of agents that can lead to hearing intoxication, patients referred for back surgery, non-smokers, without drugs, alcohol abuse and psychotropic drugs and non-allergic to anesthesia. A written consent was obtained from all the patients for the participation in the study and details of study were provided to all the participants. Patients unwilling to participate, those with hearing intoxication medications during and after surgery, patients with serious complications after surgery and reduced level of consciousness along with those who did not meet inclusion criteria were excluded from the study.

Prior to the surgery, the researcher completed a demographic questionnaire for all the participant. One audiologist was referred to the hospital admission ward who performed standard PTA and tympanometry tests using AZ80 and Madsen tofellax tympanometers and findings related to the presence or absence of hearing loss, its magnitude, frequencies, and tympanometric types were recorded in the questionnaire.

Preoperative blood pressure and other vitals were recorded for all the patients. Intraoperative monitoring devices included pulse oximeter, non-invasive blood pressure and ECG (electrocardiography) and baseline values were recorded. 2 ml/kg of 0.9% saline was administered intravenously and 27-gauge needle was used to administer 2.5 ml bupivacaine 0.5% hyperbaric using lateral approach at L2-L3 or L4-L5 slowly for 10 s at minimum.

After anesthesia, the patient was turned into supine position. Twenty minutes after anesthesia, surgery was commenced, before which sensory and motor blockade was accessed using pinprick test and Bromage scale, respectively. Blood pressure, heart rate, and pulse oximetry were measured every 15 min during the surgery. If systolic blood pressure was above 90 mmHg, 5–10 mg of intravenous ephedrine was administered to the patient. Twelve hours after surgery, the audiometry was performed again by the same audiologist. Hearing frequencies were classified as follows:

• 125–2000 Hz = (Low) low frequency
• 2000–4000 Hz = (Mid) Medium frequency
• 4000–8000 Hz = (High) High Frequency

In this study, a data entry form was used for data collection, which consisted of two parts. The first part comprised of demographic data and the second part was for recording audiometric data. This section included 7 questions including age, sex, type of surgery, MAP (mean arterial pressure), PR (pulse rate) changes above 30% baseline, amount of cerebrospinal fluid injected, and fasting time. Part II comprised of information related to the presence or absence of hearing loss and its magnitude, frequencies, and tympanometric types.

The research was approved by Ethical Committee of Lorestan University of Medical Sciences. The research was conducted in accordance with the Declaration of Helsinki (1964) and subsequent revisions. The work has been reported in line with the STROCSS criteria [24].

The collected data were analyzed by the means of descriptive statistics and analysis of variance for factorial design using SPSS 21 software. Descriptive statistics were used to summarize demographic data of the participants. Chi-square test was used to compare baseline and demographic data. Relative frequency of each case was obtained. P < 0.05 was considered significant. This study was approved by the Research Ethics Board of (XX) University of Medical Sciences (XX).

## 3. results

3

In this study, 60 patients were evaluated for the auditory function who underwent elective surgery under bupivacaine (5% marcain). The mean age of patients was 37.78 ± 8.2 years (minimum 20 years and maximum 45 years). Thirty (50%) patients were male and 30 (50%) were female. None of the patients had a history of previous gastrointestinal illness or history of substance abuse. Anesthesia was performed in 26 patients (43.30%) at L3-L4 level and in 34 patients (56.7%) at L4-L5.

Table 1 compares the right ear threshold of the studied patients before and after surgery. Results from paired T test showed that at 1000 Hz, 2000 Hz, 3000 Hz, 4000 Hz and 8000 Hz the mean difference in hearing threshold before and after hearing threshold surgery was statistically significant p = 0.007, p = 0.01 p = 0.006 and p = 0.009, respectively. The threshold was marked with a significant decrease after the surgery. However, there was no statistically significant difference in the mean threshold value of patients' right ear at frequencies 250 Hz (p = 0.077) and 500 Hz (p = 0.779) and 6000 Hz (p = 0.659) (Table 1).

**Table 1:** Comparison of mean and standard deviation of right ear hearing threshold in patients studied before and after surgery.

Threshold	After surgery μ ± SD	Before surgery μ ± SD	Mean diff	P – value
250 HZ	7.9 ± 18.25	5.69 ± 19.58	0.74 ± 1.33	0.077
500HZ	8.65 ± 17.16	17.41 ± 7.15	0.25 ± 0.18	0.779
1000HZ	8.85 ± 12.41	14.41 ± 7.25	0.74 ± 2	0.009
2000HZ	10.41 ± 11.91	9.32 ± 13.75	0.65 ± 1.83	0.007
3000HZ	16.16 ± 14.9	18.41 ± 15.55	0.84 ± 2.25	0.01
4000HZ	16.97 ± 19.66	17.65 ± 21.83	0.76 ± 2.16	0.006
6000HZ	20.86 ± 25.66	26 ± 19.52	0.15 ± 0.33	0.659
8000HZ	14.27 ± 15.25	18 ± 16.57	1.01 ± 2.75	0.009

Paired *t*-test results showed that difference in mean left ear threshold of patients before and after surgery at frequencies 250 Hz (p = 0.085), 1000 Hz (p = 0.096), 3000 Hz (p = 0.704), 6000 Hz (p = 0.38) and 8000 Hz (p = 0.349) were not statistically significant. But based on paired *t*-test results, the difference in mean values of left ear threshold of patients before and after surgery at 500 Hz (p = 0.033), 2000 Hz (p = 0.018) and 4000 Hz (p = 0.002) was statistically significant, showing that the threshold at these frequencies decreased significantly after the surgery (Table 2).

**Table 2:** Comparison of mean and standard deviation of left ear hearing threshold in patients studied before and after surgery.

Threshold	After surgery μ ± SD	Before surgery μ ± SD	Mean diff	P – value
250 HZ	16.91 ± 9.43	18 ± 9.12	1.08 ± 0.61	0.085
500HZ	14.5 ± 8.52	16.33 ± 8.17	1.83 ± 0.84	0.033
1000HZ	12.75 ± 9.97	13.75 ± 9.5	1 ± 0.59	0.096
2000HZ	14.33 ± 10.94	16.06 ± 11.04	1.73 ± 0.71	0.018
3000HZ	18.33 ± 13.42	18.58 ± 13.84	0.25 ± 0.15	0.704
4000HZ	18.91 ± 14.9	21.16 ± 13.35	2.25 ± 0.7	0.002
6000HZ	23.55 ± 16.63	24.32 ± 15.87	0.76 ± 0.56	0.38
8000HZ	18.16 ± 16.69	19.25 ± 16.22	1.08 ± 1.14	0.349

According to the T-test, the difference in the right and left ear threshold before surgery was not significantly different among males and females in any of the sexes, respectively.

Based on the results of independent T-test, the preoperative hearing threshold in the studied patients was not statistically significant at any frequency. Similarly, preoperative hearing threshold was also not statistically different at different frequencies in left and right ears, respectively.

Based on the results of the T-test, the difference in the level of right ear hearing threshold of patients after surgery was not statistically significant at any of the thresholds.

According to the results from T-test, the difference in the level of hearing loss in the left and right ear of the patients after surgery was not statistically significant (p > 0.05), respectively.

The difference in left auditory threshold was not statistically significant in any of the thresholds before surgery.

According to the results from Friedman test, the difference in mean MAP values > 30% of baseline in was statistically significant (p = 0.009) (Table 3). Similarly, the difference in mean PR values > 30% of baseline (every 15 min) was statistically significant (P < 0.001) (Table 4).

**Table 3:** Comparison of mean MAP changes of more than 30% baseline in the studied patients during the time of surgery.

MAP>30% Time 1	MAP>30% Time 2	MAP>30% Time 3	MAP>30% Time 4	Mean Rank	P-value
8.56	9:75	10	9.91	Time 1 : 2.35 Time 2 : 2.48 Time 3 : 2.58 Time 4 : 2.58	0.009

**Table 4:** Comparison of mean PR changes of more than 30% baseline (every 15 min) in patients studied over time.

MAP>30% Time 1	MAP>30% Time 2	MAP>30% Time 3	MAP>30% Time 4	Mean Rank	P-value
21.16	24.4	20.93	20.36	Time 1 : 2.78 Time 2 : 2.61 Time 3 : 2.32 Time 4 : 2.3	<0.001

According to Pearson correlation coefficients, no correlation was found between the age of patients and their hearing threshold before and after the surgery at different frequencies (p > 0.05), respectively.

Based on Spearman correlation coefficient values, no significant correlation was found between MAP values > 30% at different time interval during surgery and hearing threshold at different frequencies (p > 0.05). No significant relationship was found between values of PR changes above 30% baseline at different time intervals during surgery and patients' hearing threshold at different frequencies (p > 0.05).

According to the paired *t*-test results, the difference between the preoperative and postoperative hearing response time was not statistically significant (p = 0.702).

Based on the results of independent *t*-test, the difference in the mean of auditory reaction time of patients before the surgery was also not statistically significant (p = 0.945). This was also not significantly different in the two gender groups (p = 0.835).

Based on the results of independent *t*-test, the difference in the mean of auditory reaction time before anesthesia was not statistically significant (p = 0.904). Furthermore, age and auditory response time before surgery were not correlated significantly (p = 0.447). But according to Pearson correlation coefficients, there was a significant direct and linear relationship between age and auditory response time after surgery (p = 0.012) (Table 5).

**Table 5:** Solidarity matrix Determining the relationship between age and auditory response time before surgery in patients studied.

Type of variable	Age
Listening time before surgery	r = 0.1 pv = 0.447
Auditory response time after surgery	r = 0.324 pv = 0.012

According to Spearman correlation coefficient, preoperative auditory reaction time was not correlated with PR changes above 30% at different time intervals during the surgery.

Similarly, preoperative auditory reaction time and MAP changes> 30% of baseline at different time intervals during the surgery was also not significantly related.

## 4 Discussion

The overall results of this study show that at certain frequencies, hearing loss occurs in both the ears after spinal anesthesia, which is not related to age, sex, or level of spinal anesthesia. The results also showed that changes in mean arterial blood pressure (MAP) and heart rate above 30% of the baseline levels is not associated with the hearing loss. At low frequencies of 250 Hz and 500 Hz, hearing thresholds did not differ in the right ear, but at frequencies above 500 Hz, the hearing threshold was significantly reduced after surgery.

In a study conducted at Freeman Hospital in the Department of Otolaryngology, low frequency hearing loss was reported in patients with spinal anesthesia [25]. In the Norooznia study, hearing loss was 92% at low frequencies and hearing loss in patients was about 15–25 dB, which was consistent with the results of a study conducted at Freeman Hospital [26].

In the left ear at 250, 1000, 3000, and 8000 Hz, there was no difference in mean pre-operative hearing threshold in the right ear, and the hearing loss did not follow a specific pattern. A study reported in the anesthesiology department of Tollohelsinki Hospital, Finland, reported a reduction in the hearing ability as the risk of spinal anesthesia [27]. Acute changes in blood volume and blood pressure, intracranial pressure, and osmolarity can stimulate hearing loss [28].

In a study by Schaffartzik et al. comparing hearing loss after spinal and general anesthesia, it was concluded that hearing loss after spinal anesthesia is greater. In this study, a specific relationship was found between intravascular volume changes and low frequency of hearing loss after spinal anesthesia [29]. In another study performed at a university hospital in Phase III, unilateral vestibular dysfunction was established following spinal anesthesia [13].

The results of this study showed that pre-operative hearing threshold was not different between men and women and post-operative hearing threshold was not different between the two genders. The results of this study also showed that the preoperative hearing reaction time of patients was similar among the two genders and there was no significant difference in post-operative hearing time after the surgery. This showed that gender did not affect the hearing response time of patients after spinal anesthesia with marcaine 5%. In the study of Maleki et al. there was a significant difference between the two genders in terms of word recognition score in silence and in 3 signal-to-noise ratios. In this comparison, the mean breakthrough thresholds at the four severity levels were similar for the two groups. This means that gender is unaffected by the temporal resolution ability of individuals. Similar outcomes have been obtained from the studies by Lotze et al. [30]. and Kelso et,al [31]. According to Pearson correlation coefficients, no correlation was found between patients' age and their hearing threshold before and after the surgery at different frequencies. The results also showed that patients' age had no linear relationship with preoperative and postoperative auditory reaction time. In the study of Maleki et al. [32], Helfer et al. [33], and Dubno et al. [34], individuals in the age range of 41–55 years had weaker auditory response in the presence of noise than those in the age group of 25–40 years. Helfer et al. reported that middle-aged people have greater hearing difficulties in real-life hearing than younger people. The results of this study showed minor age-related changes in hearing processing in middle-aged people [33].

Fitzgibbons and his colleagues studied the effect of age on long-term auditory differentiation. The results concluded that short-distance differentiation was influenced by age and hearing [35]. In general, it can be said that the auditory function of people deteriorated with age. Therefore, age is an important factor in the process of hearing loss. However, our study results show that there is no relationship between age and hearing loss following spinal anesthesia.

The results of this study showed that there was no difference in right and left ear threshold in two levels of L3-L4 and L4-L5 anesthesia before surgery, indicating that the level of anesthesia has no effect on reduction of postoperative hearing level. The results showed that auditory reaction time was similar in the patients before and after anesthesia and there was no significant difference after surgery. We did not find any study addressing the level of anesthesia and its effect on hearing loss, but various studies have shown that spine surgery in different lumbar vertebrae can cause hearing loss in both right and left ears. These include the use of nitrous oxide and CSF leakage. CSF leakage is an important factor in hearing loss following spinal anesthesia because it causes brain tissue to stretch and impacts cochlear vestibular nerve and traction inside the internal acoustic crater, leading to hearing impairment.

Our study does not compare the effects of different doses and types of spinal anesthesia on hearing loss. Additionally, the data of the study is restricted to a relatively smaller sample size and is generalized for all elective surgeries (excluding back and ear). Future multicenter studies with more variables and greater sample size can give better conclusions.

## 5 Conclusion

The results show that at certain frequencies, hearing loss in both ears is observed after spinal anesthesia with 5% Marcaine, but this hearing loss is not related to age, sex and the level of spinal anesthesia. The results also showed that changes in mean arterial blood pressure (MAP) and heart rate greater than 30% of baseline is not associated with the hearing loss.

## Ethical approval

All procedures performed in this study involving human participants were in accordance with the ethical standards of the institutional and/or national research committee and with the 1964 Helsinki Declaration and its later amendments or comparable ethical standards.

## Funding source

No funding was secured for this study.

## Author contribution

Sepideh Vahabi: Conceptualization, Writing - original draft, Writing - review & editing, Conceptualized and designed the study, drafted the initial manuscript, and reviewed and revised the manuscript, Parvin Veiskarami: Writing - original draft, Writing - review & editing, Designed the data collection instruments, collected data, carried out the initial analyses, and reviewed and revised the manuscript, Mehdi Roozbahani: Writing - original draft, Writing - review & editing, Supervision, Coordinated and supervised data collection, and critically reviewed the manuscript for important intellectual content, Shahrzad Lashani: Writing - original draft, Writing - review & editing, Designed the data collection instruments, collected data, carried out the initial analyses, and reviewed and revised the manuscript, Behrouz Farzan: Conceptualization, Writing - original draft, Writing - review & editing, conceptualized and designed the study, drafted the initial manuscript, and reviewed and revised the manuscript.

## Registration of research studies

Name of the registry: Lorestan University of Medical Sciences

Unique Identifying number or registration ID: IR.LUMS.REC.1398.046.

Hyperlink to the registration (must be publicly accessible): https://ethics.research.ac.ir/ProposalCertificate.php?id=65108&Print=true&NoPrintHeader=true&NoPrintFooter=true&NoPrintPageBorder=true&LetterPrint=true

## Guarantor

Sepideh Vahabi

## Consent

Not applicable.

## Availability of data and material

Data sharing is not applicable to this article as no datasets were generated or analyzed during the current study.

## Provenance and peer review

Not commissioned, externally peer reviewed.

## Declaration of competing interest

The authors deny any conflict of interest in any terms or by any means during the study.
